# Extensions of interpolation between the arithmetic-geometric mean inequality for matrices

**DOI:** 10.1186/s13660-017-1485-x

**Published:** 2017-09-07

**Authors:** Mojtaba Bakherad, Rahmatollah Lashkaripour, Monire Hajmohamadi

**Affiliations:** Department of Mathematics, Faculty of Mathematics, University of Sistan and Baluchestan, Zahedan, Iran

**Keywords:** 47A64, 15A60, arithmetic-geometric mean, unitarily invariant norm, Hilbert-Schmidt norm, Cauchy-Schwarz inequality

## Abstract

In this paper, we present some extensions of interpolation between the arithmetic-geometric means inequality. Among other inequalities, it is shown that if *A*, *B*, *X* are $n\times n$ matrices, then

$$\begin{aligned} \bigl\Vert AXB^{*} \bigr\Vert ^{2}\leq \bigl\Vert f_{1} \bigl(A^{*}A\bigr)Xg_{1}\bigl(B^{*}B\bigr) \bigr\Vert \bigl\Vert f_{2}\bigl(A^{*}A\bigr)Xg_{2}\bigl(B^{*}B\bigr) \bigr\Vert , \end{aligned}$$

where $f_{1}$, $f_{2}$, $g_{1}$, $g_{2}$ are non-negative continuous functions such that $f_{1}(t)f_{2}(t)=t$ and $g_{1}(t)g_{2}(t)=t$ ($t\geq0$). We also obtain the inequality

$$\begin{aligned} \bigl\vert \!\bigl\vert \!\bigl\vert AB^{*} \bigr\vert \!\bigr\vert \!\bigr\vert ^{2} &\leq \bigl\vert \!\bigl\vert \!\bigl\vert p \bigl(A^{*}A\bigr)^{\frac{m}{p}}+ (1-p) \bigl(B^{*}B\bigr)^{\frac {s}{1-p}} \bigr\vert \!\bigr\vert \!\bigr\vert \bigl\vert \!\bigl\vert \!\bigl\vert (1-p) \bigl(A^{*}A\bigr)^{\frac{n}{1-p}}+ p\bigl(B^{*}B \bigr)^{\frac{t}{p}} \bigr\vert \!\bigr\vert \!\bigr\vert , \end{aligned}$$

in which *m*, *n*, *s*, *t* are real numbers such that $m+n=s+t=1$, $\vert \!\vert \!\vert \cdot \vert \!\vert \!\vert $ is an arbitrary unitarily invariant norm and $p\in[0,1]$.

## Introduction and preliminaries

Let $\mathcal{M}_{n}$ be the $C^{*}$-algebra of all $n\times n$ complex matrices and $\langle\cdot,\cdot\rangle$ be the standard scalar product in $\mathcal{C}^{n}$ with the identity *I*. The Gelfand map $f(t)\mapsto f(A)$ is an isometrical ∗-isomorphism between the $C^{*}$-algebra $C(\operatorname{sp}(A))$ of continuous functions on the spectrum $\operatorname{sp}(A)$ of a Hermitian matrix *A* and the $C^{*}$-algebra generated by *A* and *I*.

A norm $\vert \!\vert \!\vert \cdot \vert \!\vert \!\vert $ on $\mathcal{M}_{n}$ is said to be unitarily invariant norm if $\vert \!\vert \!\vert UAV \vert \!\vert \!\vert = \vert \!\vert \!\vert A \vert \!\vert \!\vert $, for all unitary matrices *U* and *V*. For $A\in\mathcal{M}_{n}$, let $s_{1}(A) \geq s_{2}(A) \geq\cdots \geq s_{n}(A)$ denote the singular values of *A*, *i.e.* the eigenvalues of the positive semidefinite matrix $\vert A \vert = (A^{*}A)^{\frac{1}{2}}$ arranged in a decreasing order with their multiplicities counted. Note that $s_{j}(A)=s_{j}(A^{*})=s_{j}( \vert A \vert )$ ($1\leq j \leq n$) and $\Vert A \Vert =s_{1}(A)$. The Ky Fan norm of a matrix *A* is defined as $\Vert A \Vert _{(k)}=\sum_{j=1}^{k} s_{j}(A)$ ($1\leq k\leq n$). The Fan dominance theorem asserts that $\Vert A \Vert _{ (k)} \leq \Vert B \Vert _{(k)}$ for $k = 1, 2, \ldots, n$ if and only if $\vert \!\vert \!\vert A \vert \!\vert \!\vert \leq \vert \!\vert \!\vert B \vert \!\vert \!\vert $ for every unitarily invariant norm(see [1], p.93). The Hilbert-Schmidt norm is defined by $\Vert A \Vert _{2}= (\sum_{j=1}^{n}s_{j}^{2}(A) )^{1/2}$, which is unitarily invariant.

The classical Cauchy-Schwarz inequality for $a_{j}\geq0$, $b_{j}\geq0$ ($1\leq j\leq n$) states that

$$\begin{aligned} \Biggl(\sum_{j=1}^{n} a_{j}b_{j} \Biggr)^{2}\leq \Biggl(\sum_{j=1}^{n} a_{j}^{2} \Biggr) \Biggl(\sum_{j=1}^{n} b_{j}^{2} \Biggr) \end{aligned}$$

with equality if and only if $(a_{1},\ldots, a_{n})$ and $(b_{1},\ldots, b_{n})$ are proportional [2]. Bhatia and Davis gave a matrix Cauchy-Schwarz inequality as follows:

1 $$\begin{aligned} \bigl\vert \!\bigl\vert \!\bigl\vert AXB^{*} \bigr\vert \!\bigr\vert \!\bigr\vert ^{2}\leq \bigl\vert \!\bigl\vert \!\bigl\vert A^{*}AX \bigr\vert \!\bigr\vert \!\bigr\vert \bigl\vert \!\bigl\vert \!\bigl\vert XB^{*}B \bigr\vert \!\bigr\vert \!\bigr\vert , \end{aligned}$$

where $A, B, X\in\mathcal{M}_{n}$. For further information as regards the Cauchy-Schwarz inequality, see [3–5] and the references therein. Recently, Kittaneh *et al.* [6] extended inequality (1) to the form

2 $$\begin{aligned} \bigl\vert \!\bigl\vert \!\bigl\vert AXB^{*} \bigr\vert \!\bigr\vert \!\bigr\vert ^{2}\leq \bigl\vert \!\bigl\vert \!\bigl\vert \bigl(A^{*}A\bigr)^{p}X\bigl(B^{*}B\bigr)^{1-p} \bigr\vert \!\bigr\vert \!\bigr\vert \bigl\vert \!\bigl\vert \!\bigl\vert \bigl(A^{*}A\bigr)^{1-p}X\bigl(B^{*}B\bigr)^{p} \bigr\vert \!\bigr\vert \!\bigr\vert , \end{aligned}$$

where $A, B, X\in\mathcal{M}_{n}$ and $p\in[0,1]$. Audenaert [7] proved that, for all $A, B\in\mathcal{M}_{n}$ and all $p \in[0, 1]$, we have

3 $$\begin{aligned} \bigl\vert \!\bigl\vert \!\bigl\vert AB^{*} \bigr\vert \!\bigr\vert \!\bigr\vert ^{2}\leq \bigl\vert \!\bigl\vert \!\bigl\vert pA^{*}A+(1-p)B^{*}B \bigr\vert \!\bigr\vert \!\bigr\vert \bigl\vert \!\bigl\vert \!\bigl\vert (1-p)A^{*}A+pB^{*}B \bigr\vert \!\bigr\vert \!\bigr\vert . \end{aligned}$$

In [8], the authors generalized inequality (3) for all $A, B, X\in\mathcal{M}_{n}$ and all $p \in[0, 1]$ to the form

4 $$\begin{aligned} \bigl\vert \!\bigl\vert \!\bigl\vert AXB^{*} \bigr\vert \!\bigr\vert \!\bigr\vert ^{2}\leq \bigl\vert \!\bigl\vert \!\bigl\vert pA^{*}AX+(1-p)XB^{*}B \bigr\vert \!\bigr\vert \!\bigr\vert \bigl\vert \!\bigl\vert \!\bigl\vert (1-p)A^{*}AX+pXB^{*}B \bigr\vert \!\bigr\vert \!\bigr\vert . \end{aligned}$$

Inequality (4) interpolates between the arithmetic-geometric mean inequality. In [6], the authors showed a refinement of inequality (4) for the Hilbert-Schmidt norm as follows:

5 $$\begin{aligned} \bigl\Vert AXB^{*} \bigr\Vert _{2}^{2}&\leq \bigl( \bigl\Vert p A^{*}A X+(1-p) XB^{*}B \bigr\Vert _{2}^{2} -r^{2} \bigl\Vert A^{*}AX-XB^{*}B \bigr\Vert _{2}^{2} \bigr) \\ & \quad{}\times \bigl( \bigl\Vert (1-p) A^{*}A X+p XB^{*}B \bigr\Vert _{2}^{2} -r^{2} \bigl\Vert A^{*}AX-XB^{*}B \bigr\Vert _{2}^{2} \bigr), \end{aligned}$$

in which $A, B, X\in\mathcal{M}_{n}$, $p\in[0,1]$ and $r=\min\{p,1-p\}$. The Young inequality for every unitarily invariant norm states that $\vert \!\vert \!\vert A^{p}B^{1-p} \vert \!\vert \!\vert \leq \vert \!\vert \!\vert pA+(1-p)B \vert \!\vert \!\vert $, where *A*, *B* are positive definite matrices and $p\in[0,1]$ (see [9] and also [10, 11]). Kosaki [12] extended the last inequality for the Hilbert-Schmidt norm as follows:

6 $$\begin{aligned} \bigl\Vert A^{p}XB^{1-p} \bigr\Vert _{2}\leq \bigl\Vert pAX+(1-p)XB \bigr\Vert _{2}, \end{aligned}$$

where *A*, *B* are positive definite matrices, *X* is any matrix and $p\in[0,1]$. In [13], the authors considered as a refined matrix Young inequality for the Hilbert-Schmidt norm

7 $$\begin{aligned} \bigl\Vert A^{p}XB^{1-p} \bigr\Vert _{2}^{2}+r^{2} \Vert AX-XB \Vert _{2}^{2}\leq \bigl\Vert pAX+(1-p)XB \bigr\Vert _{2}^{2}, \end{aligned}$$

in which *A*, *B* are positive semidefinite matrices, $X\in\mathcal {M}_{n}$, $p\in[0,1]$ and $r=\min\{p,1-p\}$.

Based on the refined Young inequality (7), Zhao and Wu [14] proved that

8 $$\begin{aligned} \bigl\Vert A^{p}XB^{1-p} \bigr\Vert _{2}^{2}+r_{0} \bigl\Vert A^{\frac {1}{2}}XB^{\frac{1}{2}}-AX \bigr\Vert _{2}^{2}+(1-p)^{2} \Vert AX-XB \Vert _{2}^{2}\leq \bigl\Vert pAX+(1-p)XB \bigr\Vert _{2}^{2}, \end{aligned}$$

for $0< p\leq\frac{1}{2}$ and

$$\begin{aligned} \bigl\Vert A^{p}XB^{1-p} \bigr\Vert _{2}^{2}+r_{0} \bigl\Vert A^{\frac {1}{2}}XB^{\frac{1}{2}}-XB \bigr\Vert _{2}^{2}+p^{2} \Vert AX-XB \Vert _{2}^{2}\leq \bigl\Vert pAX+(1-p)XB \bigr\Vert _{2}^{2}, \end{aligned}$$

for $\frac{1}{2}< p<1$ such that $r=\min\{p, 1-p\}$ and $r_{0}=\min\{ 2r, 1-2r\}$.

In this paper, we obtain some operator and unitarily invariant norms inequalities. Among other results, we obtain a refinement of inequality (5) and we also extend inequalities (2), (3) and (5) to the function $f(t)=t^{p}$ ($p\in\mathcal{R}$).

## Main results

In this section, using some ideas of [6, 15], we extend the Audenaert results for the operator norm.

### Theorem 1

*Let* *A*, *B*, $X\in\mathcal{M}_{n}$ *and* $f_{1}$, $f_{2}$, $g_{1}$, $g_{2}$ *be non*-*negative continuous functions such that* $f_{1}(t)f_{2}(t)=t$ *and* $g_{1}(t)g_{2}(t)=t$ ($t\geq0$). *Then*

9 $$\begin{aligned} \bigl\Vert AXB^{*} \bigr\Vert ^{2}\leq \bigl\Vert f_{1}\bigl(A^{*}A\bigr)Xg_{1}\bigl(B^{*}B\bigr) \bigr\Vert \bigl\Vert f_{2}\bigl(A^{*}A\bigr)Xg_{2}\bigl(B^{*}B\bigr) \bigr\Vert . \end{aligned}$$

### Proof

It follows from

$$\begin{aligned} \bigl\Vert AXB^{*} \bigr\Vert ^{2} =& \bigl\Vert BX^{*}A^{*}AXB^{*} \bigr\Vert \\ =&s_{1} \bigl(BX^{*}A^{*}AXB^{*} \bigr) \\ =&\lambda_{\max} \bigl(BX^{*}A^{*}AXB^{*} \bigr)\quad \bigl(\text{since }BX^{*}A^{*}AXB^{*} \text{ is positive semidefinite}\bigr) \\ =& \lambda_{\max} \bigl(X^{*}A^{*}AXB^{*}B \bigr) \\ =&\lambda_{\max} \bigl(X^{*}f_{1}\bigl(A^{*}A \bigr)f_{2}\bigl(A^{*}A\bigr)Xg_{2}\bigl(B^{*}B \bigr)g_{1}\bigl(B^{*}B\bigr) \bigr) \\ =&\lambda_{\max} \bigl(g_{1}\bigl(B^{*}B\bigr)X^{*}f_{1} \bigl(A^{*}A\bigr)f_{2}\bigl(A^{*}A\bigr)Xg_{2}\bigl(B^{*}B\bigr) \bigr) \\ \leq& \bigl\Vert g_{1}\bigl(B^{*}B\bigr)X^{*}f_{1}\bigl(A^{*}A \bigr)f_{2}\bigl(A^{*}A\bigr)Xg_{2}\bigl(B^{*}B\bigr) \bigr\Vert \\ \leq& \bigl\Vert g_{1}\bigl(B^{*}B\bigr)X^{*}f_{1}\bigl(A^{*}A \bigr) \bigr\Vert \bigl\Vert f_{2}\bigl(A^{*}A\bigr)Xg_{2} \bigl(B^{*}B\bigr) \bigr\Vert \\ =& \bigl\Vert f_{1}\bigl(A^{*}A\bigr)Xg_{1}\bigl(B^{*}B \bigr) \bigr\Vert \bigl\Vert f_{2}\bigl(A^{*}A\bigr)Xg_{2} \bigl(B^{*}B\bigr) \bigr\Vert \end{aligned}$$

that we get the desired result. □

### Corollary 2

*If* *A*, *B*, $X\in\mathcal{M}_{n}$ *and* *m*, *n*, *s*, *t* *are real numbers such that* $m+n=s+t=1$, *then*

10 $$\begin{aligned} \bigl\Vert AXB^{*} \bigr\Vert ^{2}\leq \bigl\Vert \bigl(A^{*}A\bigr)^{m}X\bigl(B^{*}B\bigr)^{s} \bigr\Vert \bigl\Vert \bigl(A^{*}A\bigr)^{n}X\bigl(B^{*}B\bigr)^{t} \bigr\Vert . \end{aligned}$$

In the next results, we show some generalizations of inequality (3) for the operator norm.

### Corollary 3

*Let* $A, B\in\mathcal{M}_{n}$ *and let* $f_{1}$, $f_{2}$, $g_{1}$, $g_{2}$ *be non*-*negative continuous functions such that* $f_{1}(t)f_{2}(t)=t$ *and* $g_{1}(t)g_{2}(t)=t$ ($t\geq0$). *Then*

$$\begin{aligned} \bigl\Vert AB^{*} \bigr\Vert ^{2}\leq \bigl\Vert pf_{1} \bigl(A^{*}A\bigr)^{\frac{1}{p}}+(1-p)g_{1}\bigl(B^{*}B \bigr)^{\frac {1}{1-p}} \bigr\Vert \bigl\Vert (1-p) f_{2} \bigl(A^{*}A\bigr)^{\frac {1}{1-p}}+pg_{2}\bigl(B^{*}B \bigr)^{\frac{1}{p}} \bigr\Vert , \end{aligned}$$

*where* $p\in[0,1]$.

### Proof

Applying Theorem 1 for $X=I$, we have

$$\begin{aligned} \bigl\Vert AB^{*} \bigr\Vert ^{2} \leq& \bigl\Vert f_{1}\bigl(A^{*}A\bigr) g_{1}\bigl(B^{*}B\bigr) \bigr\Vert \bigl\Vert f_{2}\bigl(A^{*}A\bigr) g_{2}\bigl(B^{*}B\bigr) \bigr\Vert \\ =& \bigl\Vert \bigl(f_{1}\bigl(A^{*}A\bigr)^{\frac{1}{p}} \bigr)^{p} \bigl( g_{1}\bigl(B^{*}B\bigr)^{\frac {1}{1-p}} \bigr)^{1-p} \bigr\Vert \bigl\Vert \bigl(f_{2} \bigl(A^{*}A\bigr)^{\frac {1}{1-p}} \bigr)^{1-p} \bigl( g_{2}\bigl(B^{*}B\bigr)^{\frac{1}{p}} \bigr)^{p} \bigr\Vert \\ & (\text{by Theorem 1)} \\ \leq& \bigl\Vert pf_{1}\bigl(A^{*}A\bigr)^{\frac{1}{p}}+(1-p)g_{1}\bigl(B^{*}B\bigr)^{\frac {1}{1-p}} \bigr\Vert \bigl\Vert (1-p) f_{2}\bigl(A^{*}A\bigr)^{\frac {1}{1-p}}+pg_{2}\bigl(B^{*}B\bigr)^{\frac{1}{p}} \bigr\Vert \\ & (\text{by the Young inequality}). \end{aligned}$$

 □

### Corollary 4

*Let* $A, B\in\mathcal{M}_{n}$ *and let* *f*, *g* *be non*-*negative continuous functions such that* $f(t)g(t)=t^{2}$ ($t\geq0$). *Then*

$$\begin{aligned} \bigl\Vert AB^{*} \bigr\Vert ^{2} \leq& \bigl\Vert p f\bigl(A^{*}A \bigr) +(1-p) g\bigl(B^{*}B\bigr) \bigr\Vert ^{\frac{1}{2}} \bigl\Vert (1-p) f\bigl(A^{*}A\bigr) +p g\bigl(B^{*}B\bigr) \bigr\Vert ^{\frac{1}{2}} \\ &{}\times \bigl\Vert p g\bigl(A^{*}A\bigr)+(1-p)f\bigl(B^{*}B\bigr) \bigr\Vert ^{\frac{1}{2}} \bigl\Vert (1-p)g\bigl(A^{*}A\bigr)+p f\bigl(B^{*}B\bigr) \bigr\Vert ^{\frac{1}{2}}, \end{aligned}$$

*where* $p\in[0,1]$.

### Proof

Applying Theorem 1 and the Young inequality we get

$$\begin{aligned} \bigl\Vert AB^{*} \bigr\Vert ^{4} \leq& \bigl\Vert f\bigl(A^{*}A \bigr)^{\frac{1}{2}} g\bigl(B^{*}B\bigr)^{\frac{1}{2}} \bigr\Vert ^{2} \bigl\Vert g\bigl(A^{*}A\bigr)^{\frac{1}{2}} f\bigl(B^{*}B \bigr)^{\frac{1}{2}} \bigr\Vert ^{2} \\ & (\text{by Theorem 1 for} \sqrt{f} \text{ and } \sqrt{g}) \\ \leq& \bigl\Vert f\bigl(A^{*}A\bigr)^{p} g\bigl(B^{*}B \bigr)^{1-p} \bigr\Vert \bigl\Vert f\bigl(A^{*}A\bigr)^{1-p} g \bigl(B^{*}B\bigr)^{p} \bigr\Vert \\ &{}\times \bigl\Vert g\bigl(A^{*}A\bigr)^{p} f\bigl(B^{*}B \bigr)^{1-p} \bigr\Vert \bigl\Vert g\bigl(A^{*}A\bigr)^{1-p} f \bigl(B^{*}B\bigr)^{p} \bigr\Vert \\ & (\text{by inequality (10)}) \\ \leq& \bigl\Vert p f\bigl(A^{*}A\bigr)+(1-p)g\bigl(B^{*}B\bigr) \bigr\Vert \bigl\Vert (1-p)f\bigl(A^{*}A\bigr)+p g\bigl(B^{*}B\bigr) \bigr\Vert \\ &{}\times \bigl\Vert p g\bigl(A^{*}A\bigr)+(1-p)f\bigl(B^{*}B\bigr) \bigr\Vert \bigl\Vert (1-p)g\bigl(A^{*}A\bigr)+p f\bigl(B^{*}B\bigr) \bigr\Vert \\ & (\text{by the Young inequality}). \end{aligned}$$

 □

## Some interpolations for unitarily invariant norms

In this section, applying some ideas of [6], we generalize some interpolations for an arbitrary unitarily invariant norm.

Let $Q_{k,n}$ denote the set of all strictly increasing *k*-tuples chosen from ${1, 2,\ldots, n}$, *i.e.* $I \in Q_{k,n}$ if $I = (i_{1}, i_{2}, \ldots, i_{k})$, where $1\leq i_{1}< i_{2}<\cdots< i_{k}\leq n$. The following lemma gives some properties of the *k*th antisymmetric tensor powers of matrices in $\mathcal{M}_{n}$; see [1], p.18.

### Lemma 5

*Let* $A, B\in\mathcal{M}_{n}$. *Then*

- $(\bigwedge^{k}A)(\bigwedge^{k}B)=\bigwedge^{k}(AB)$ *for* $k=1,\ldots,n$.
- $(\bigwedge^{k}A)^{*}=\bigwedge^{k}A^{*}$ *for* $k=1,\ldots,n$.
- $(\bigwedge^{k}A)^{-1}=\bigwedge^{k}A^{-1}$ *for* $k=1,\ldots,n$.
- *If* $s_{1}, s_{2}, \ldots, s_{n}$ *are the singular values of* *A*, *then the singular values of* $\bigwedge^{k}A$ *are* $s_{i_{1}}, s_{i_{2}}, \ldots, s_{i_{k}}$, *where* $({i_{1}}, {i_{2}}, \ldots, {i_{k}})\in Q_{k,n}$.

Now, we show inequality (10) for an arbitrary unitarily invariant norm.

### Theorem 6

*Let* $A, B, X\in\mathcal{M}_{n}$ *and* $\vert \!\vert \!\vert \cdot \vert \!\vert \!\vert $ *be an arbitrary unitarily invariant norm*. *Then*

11 $$\begin{aligned} \bigl\vert \!\bigl\vert \!\bigl\vert AXB^{*} \bigr\vert \!\bigr\vert \!\bigr\vert ^{2}\leq \bigl\vert \!\bigl\vert \!\bigl\vert \bigl(A^{*}A\bigr)^{m}X\bigl(B^{*}B\bigr)^{s} \bigr\vert \!\bigr\vert \!\bigr\vert \bigl\vert \!\bigl\vert \!\bigl\vert \bigl(A^{*}A\bigr)^{n}X\bigl(B^{*}B\bigr)^{t} \bigr\vert \!\bigr\vert \!\bigr\vert , \end{aligned}$$

*where* *m*, *n*, *s*, *t* *are real numbers such that* $m+n=s+t=1$. *In particular*, *if* *A*, *B* *are positive definite*, *then*

12 $$\begin{aligned} \bigl\vert \!\bigl\vert \!\bigl\vert A^{\frac{1}{2}}XB^{\frac{1}{2}} \bigr\vert \!\bigr\vert \!\bigr\vert ^{2}\leq \bigl\vert \!\bigl\vert \!\bigl\vert A^{p} XB^{1-p} \bigr\vert \!\bigr\vert \!\bigr\vert \bigl\vert \!\bigl\vert \!\bigl\vert A^{1-p}XB^{p} \bigr\vert \!\bigr\vert \!\bigr\vert , \end{aligned}$$

*where* $p\in[0,1]$.

### Proof

If we replace *A*, *B* and *X* by $\bigwedge^{k}A$, $\bigwedge^{k}B$ and $\bigwedge^{k}X$, their *k*th antisymmetric tensor powers in inequality (9) and apply Lemma 5, then we have

$$\begin{aligned} \biggl\Vert \bigwedge^{k}AXB^{*} \biggr\Vert ^{2}\leq \biggl\Vert \bigwedge^{k}\bigl(A^{*}A\bigr)^{m}X\bigl(B^{*}B \bigr)^{s} \biggr\Vert \biggl\Vert \bigwedge^{k}\bigl(A^{*}A \bigr)^{n}X\bigl(B^{*}B\bigr)^{t} \biggr\Vert , \end{aligned}$$

which is equivalent to

$$\begin{aligned} s_{1}^{2} \biggl(\bigwedge^{k}AXB^{*} \biggr)\leq s_{1} \biggl(\bigwedge^{k}\bigl(A^{*}A\bigr)^{m}X \bigl(B^{*}B\bigr)^{s} \biggr) s_{1} \biggl(\bigwedge^{k} \bigl(A^{*}A\bigr)^{n}X\bigl(B^{*}B\bigr)^{t} \biggr). \end{aligned}$$

Applying Lemma 5(d), we have

13 $$\begin{aligned} \prod_{j=1}^{k}s_{j} \bigl(AXB^{*} \bigr) \leq& \prod_{j=1}^{k}s_{j}^{\frac{1}{2}} \bigl(\bigl(A^{*}A\bigr)^{m}X\bigl(B^{*}B\bigr)^{s} \bigr)\prod _{j=1}^{k}s_{j}^{\frac{1}{2}} \bigl(\bigl(A^{*}A\bigr)^{n}X\bigl(B^{*}B\bigr)^{t} \bigr) \\ \leq& \prod_{j=1}^{k}s_{j}^{\frac{1}{2}} \bigl(\bigl(A^{*}A\bigr)^{m}X\bigl(B^{*}B\bigr)^{s} \bigr)s_{j}^{\frac{1}{2}} \bigl(\bigl(A^{*}A\bigr)^{n}X \bigl(B^{*}B\bigr)^{t} \bigr), \end{aligned}$$

where $k=1,\ldots,n$. Inequality (13) implies that

$$\begin{aligned} \sum_{j=1}^{k}s_{j} \bigl(AXB^{*} \bigr) \leq& \sum_{j=1}^{k}s_{j}^{\frac {1}{2}} \bigl(\bigl(A^{*}A\bigr)^{m}X\bigl(B^{*}B\bigr)^{s} \bigr)s_{j}^{\frac{1}{2}} \bigl(\bigl(A^{*}A\bigr)^{n}X \bigl(B^{*}B\bigr)^{t} \bigr) \\ \leq& \Biggl(\sum_{j=1}^{k}s_{j} \bigl(\bigl(A^{*}A\bigr)^{m}X\bigl(B^{*}B\bigr)^{s} \bigr) \Biggr)^{\frac{1}{2}} \Biggl(\sum_{j=1}^{k}s_{j} \bigl(\bigl(A^{*}A\bigr)^{n}X\bigl(B^{*}B\bigr)^{t} \bigr) \Biggr)^{\frac{1}{2}} \\ & \text{(by the Cauchy-Schwarz inequality)}, \end{aligned}$$

where $k=1,\ldots,n$. Hence

$$\begin{aligned} \bigl\Vert AXB^{*} \bigr\Vert _{(k)}^{2}\leq \bigl\Vert \bigl(A^{*}A\bigr)^{m}X\bigl(B^{*}B\bigr)^{s} \bigr\Vert _{(k)} \bigl\Vert \bigl(A^{*}A\bigr)^{n}X\bigl(B^{*}B \bigr)^{t} \bigr\Vert _{(k)}. \end{aligned}$$

Now, using the Fan dominance theorem [1], p.98, we get the desired result. □

Now, using inequality (12), Theorem 6 and the same argument in the proof of Corollaries 3 and 4, we get the following results; these inequalities are generalizations of the Audenaert inequality (3).

### Corollary 7

*Let* $A, B\in\mathcal{M}_{n}$, *m*, *n*, *s*, *t* *be real numbers such that* $m+n=s+t=1$ *and let* $\vert \!\vert \!\vert \cdot \vert \!\vert \!\vert $ *be an arbitrary unitarily invariant norm*. *Then*

$$\begin{aligned} \bigl\vert \!\bigl\vert \!\bigl\vert AB^{*} \bigr\vert \!\bigr\vert \!\bigr\vert ^{2} &\leq \bigl\vert \!\bigl\vert \!\bigl\vert p \bigl(A^{*}A\bigr)^{\frac{m}{p}}+ (1-p) \bigl(B^{*}B\bigr)^{\frac {s}{1-p}} \bigr\vert \!\bigr\vert \!\bigr\vert \bigl\vert \!\bigl\vert \!\bigl\vert (1-p) \bigl(A^{*}A\bigr)^{\frac{n}{1-p}}+ p\bigl(B^{*}B \bigr)^{\frac{t}{p}} \bigr\vert \!\bigr\vert \!\bigr\vert , \end{aligned}$$

*where* $p\in[0,1]$.

### Corollary 8

*Let* $A, B\in\mathcal{M}_{n}$, *m*, *n*, *s*, *t* *be real numbers such that* $m+n=s+t=2$ *and let* $\vert \!\vert \!\vert \cdot \vert \!\vert \!\vert $ *be an arbitrary unitarily invariant norm*. *Then*

14 $$\begin{aligned} \bigl\vert \!\bigl\vert \!\bigl\vert AB^{*} \bigr\vert \!\bigr\vert \!\bigr\vert ^{2} &\leq \bigl\vert \!\bigl\vert \!\bigl\vert p\bigl(A^{*}A\bigr)^{m}+ (1-p) \bigl(B^{*}B\bigr)^{s} \bigr\vert \!\bigr\vert \!\bigr\vert ^{\frac{1}{2}} \bigl\vert \!\bigl\vert \!\bigl\vert (1-p) \bigl(A^{*}A \bigr)^{m}+ p\bigl(B^{*}B\bigr)^{s} \bigr\vert \!\bigr\vert \!\bigr\vert ^{\frac{1}{2}} \\ &\quad{}\times \bigl\vert \!\bigl\vert \!\bigl\vert p\bigl(A^{*}A\bigr)^{n}+ (1-p) \bigl(B^{*}B \bigr)^{t} \bigr\vert \!\bigr\vert \!\bigr\vert ^{\frac{1}{2}} \bigl\vert \!\bigl\vert \!\bigl\vert (1-p) \bigl(A^{*}A\bigr)^{n}+p\bigl(B^{*}B\bigr)^{t} \bigr\vert \!\bigr\vert \!\bigr\vert ^{\frac{1}{2}} \end{aligned}$$

*in which* $p\in[0,1]$.

### Remark 9

If we put $n=m=s=t=1$ in inequality (14), then we obtain the Audenaert inequality (3). Also, if we use inequality (6), Corollaries 7 and 8, then similar to Corollaries 3 and 4 we get the following inequalities:

15 $$\begin{aligned} \bigl\Vert AXB^{*} \bigr\Vert _{2}^{2}&\leq \bigl\Vert p\bigl(A^{*}A\bigr)^{\frac{m}{p}}X+ (1-p)X\bigl(B^{*}B \bigr)^{\frac {s}{1-p}} \bigr\Vert _{2} \\ &\quad {}\times \bigl\Vert (1-p) \bigl(A^{*}A \bigr)^{\frac{n}{1-p}}X+ pX\bigl(B^{*}B\bigr)^{\frac{t}{p}} \bigr\Vert _{2}, \end{aligned}$$

where $A, B\in\mathcal{M}_{n}$, *m*, *n*, *s*, *t* are real numbers such that $m+n=s+t=1$, $p\in[0,1]$ and

$$\begin{aligned} \bigl\Vert AXB^{*} \bigr\Vert _{2}^{2} &\leq \bigl\Vert p\bigl(A^{*}A\bigr)^{m}X+ (1-p)X\bigl(B^{*}B\bigr)^{s} \bigr\Vert _{2}^{\frac{1}{2}} \bigl\Vert (1-p) \bigl(A^{*}A \bigr)^{m}X+ pX\bigl(B^{*}B\bigr)^{s} \bigr\Vert _{2}^{\frac{1}{2}} \\ &\quad{}\times \bigl\Vert p\bigl(A^{*}A\bigr)^{n}X+ (1-p)X\bigl(B^{*}B \bigr)^{t} \bigr\Vert _{2}^{\frac{1}{2}} \bigl\Vert (1-p) \bigl(A^{*}A\bigr)^{n}X+pX\bigl(B^{*}B\bigr)^{t} \bigr\Vert _{2}^{\frac{1}{2}} \end{aligned}$$

for $A, B\in\mathcal{M}_{n}$, real numbers *m*, *n*, *s*, *t*, in which $m+n=s+t=2$ and $p\in[0,1]$. These inequalities are generalizations of (4) for the Hilbert-Schmidt norms.

In the following theorem, we show a refinement of inequality (15) for the Hilbert-Schmidt norm.

### Theorem 10

*Let* $A, B, X\in\mathcal{M}_{n}$. *Then*

$$\begin{aligned} \bigl\Vert AXB^{*} \bigr\Vert _{2}^{2}&\leq \bigl( \bigl\Vert p \bigl(A^{*}A\bigr)^{\frac{m}{p}} X+(1-p) X\bigl(B^{*}B \bigr)^{\frac{s}{1-p}} \bigr\Vert _{2}^{2} -r^{2} \bigl\Vert \bigl(A^{*}A\bigr)^{\frac{m}{p}}X-X \bigl(B^{*}B\bigr)^{\frac{s}{1-p}} \bigr\Vert _{2}^{2} \bigr) \\ & \quad {}\times \bigl( \bigl\Vert (1-p) \bigl(A^{*}A\bigr)^{\frac{n}{1-p}} X+p X\bigl(B^{*}B\bigr)^{\frac{t}{p}} \bigr\Vert _{2}^{2} -r^{2} \bigl\Vert \bigl(A^{*}A\bigr)^{\frac{n}{1-p}}X-X \bigl(B^{*}B\bigr)^{\frac{t}{p}} \bigr\Vert _{2}^{2} \bigr), \end{aligned} $$

*in which* *m*, *n*, *s*, *t* *are real numbers such that* $m+n=s+t=1$, $p\in[0,1]$ *and* $r=\min\{p,1-p\}$.

### Proof

Applying inequality (11), we deduce that

$$\begin{aligned} \bigl\Vert AXB^{*} \bigr\Vert _{2}^{2} \leq& \bigl\Vert \bigl(A^{*}A\bigr)^{m}X\bigl(B^{*}B\bigr)^{s} \bigr\Vert _{2} \bigl\Vert \bigl(A^{*}A\bigr)^{n}X\bigl(B^{*}B \bigr)^{t} \bigr\Vert _{2} \\ =& \bigl\Vert \bigl(\bigl(A^{*}A\bigr)^{\frac{m}{p}} \bigr)^{p}X \bigl(\bigl(B^{*}B\bigr)^{\frac{s}{1-p}} \bigr)^{1-p} \bigr\Vert _{2} \bigl\Vert \bigl(\bigl(A^{*}A \bigr)^{\frac{n}{1-p}} \bigr)^{1-p}X \bigl(\bigl(B^{*}B \bigr)^{\frac{t}{p}} \bigr)^{p} \bigr\Vert _{2} \\ \leq& \bigl( \bigl\Vert p \bigl(A^{*}A\bigr)^{\frac{m}{p}} X+(1-p) X \bigl(B^{*}B\bigr)^{\frac{s}{1-p}} \bigr\Vert _{2}^{2} -r^{2} \bigl\Vert \bigl(A^{*}A\bigr)^{\frac{m}{p}}X-X \bigl(B^{*}B\bigr)^{\frac{s}{1-p}} \bigr\Vert _{2}^{2} \bigr) \\ & {}\times \bigl( \bigl\Vert (1-p) \bigl(A^{*}A\bigr)^{\frac{n}{1-p}} X+p X\bigl(B^{*}B\bigr)^{\frac{t}{p}} \bigr\Vert _{2}^{2} -r^{2} \bigl\Vert \bigl(A^{*}A\bigr)^{\frac{n}{1-p}}X-X \bigl(B^{*}B\bigr)^{\frac{t}{p}} \bigr\Vert _{2}^{2} \bigr), \end{aligned}$$

where $p\in[0,1]$ and $r=\min\{p,1-p\}$, and the proof is complete. □

Theorem 10 includes a special case as follows.

### Corollary 11

[6], Theorem 2.5

*Let* $A, B, X\in\mathcal{M}_{n}$. *Then*

$$\begin{aligned} \bigl\Vert AXB^{*} \bigr\Vert _{2}^{2} \leq& \bigl( \bigl\Vert p A^{*}A X+(1-p) XB^{*}B \bigr\Vert _{2}^{2} -r^{2} \bigl\Vert A^{*}AX-XB^{*}B \bigr\Vert _{2}^{2} \bigr) \\ & {}\times \bigl( \bigl\Vert (1-p) \bigl(A^{*}A\bigr) X+p XB^{*}B \bigr\Vert _{2}^{2} -r^{2} \bigl\Vert A^{*}AX-XB^{*}B \bigr\Vert _{2}^{2} \bigr), \end{aligned}$$

*where* $p\in[0,1]$ *and* $r=\min\{p,1-p\}$.

### Proof

For $p\in[0,1]$, if we put $m=t=p$ and $n=s=1-p$ in Theorem 10, then we get the desired result. □

The next result is a refinement of inequality (5).

### Theorem 12

*Let* $A, B, X\in\mathcal{M}_{n}({\mathcal {C}})$ *and let* $p\in(0, 1)$. *Then*

(i) *For* $0< p\leq\frac{1}{2}$,
  16 $$\begin{aligned} \bigl\Vert AXB^{*} \bigr\Vert _{2}^{2}& \leq \bigl( \bigl\Vert pA^{*}AX+(1-p)XB^{*}B \bigr\Vert _{2}^{2}-r_{0} \bigl\Vert \bigl(A^{*}A\bigr)^{\frac{1}{2}}X\bigl(B^{*}B \bigr)^{\frac{1}{2}}-A^{*}AX \bigr\Vert _{2}^{2} \\ &\quad {}-(1-p)^{2} \bigl\Vert A^{*}AX-XB^{*}B \bigr\Vert _{2}^{2} \bigr)^{\frac{1}{2}} \\ &\quad {}\times \bigl( \bigl\Vert (1-p)A^{*}AX+pXB^{*}B \bigr\Vert _{2}^{2}-r_{0} \bigl\Vert \bigl(A^{*}A\bigr)^{\frac{1}{2}}X\bigl(B^{*}B \bigr)^{\frac {1}{2}}-A^{*}AX \bigr\Vert _{2}^{2} \\ &\quad {}-p^{2} \bigl\Vert A^{*}AX-XB^{*}B \bigr\Vert _{2}^{2} \bigr)^{\frac{1}{2}}. \end{aligned}$$
(ii) *For* $\frac{1}{2}< p<1$,
  17 $$\begin{aligned} \bigl\Vert AXB^{*} \bigr\Vert _{2}^{2}& \leq \bigl( \bigl\Vert pA^{*}AX+(1-p)XB^{*}B \bigr\Vert _{2}^{2}-r_{0} \bigl\Vert \bigl(A^{*}A\bigr)^{\frac{1}{2}}X\bigl(B^{*}B \bigr)^{\frac{1}{2}}-X\bigl(B^{*}B\bigr) \bigr\Vert _{2}^{2} \\ &\quad {}-(1-p)^{2} \bigl\Vert A^{*}AX-XB^{*}B \bigr\Vert _{2}^{2} \bigr)^{\frac{1}{2}} \\ &\quad {}\times \bigl( \bigl\Vert (1-p)A^{*}AX+pXB^{*}B \bigr\Vert _{2}^{2}-r_{0} \bigl\Vert \bigl(A^{*}A\bigr)^{\frac{1}{2}}X\bigl(B^{*}B \bigr)^{\frac {1}{2}}-X\bigl(B^{*}B\bigr) \bigr\Vert _{2}^{2} \\ &\quad {}-p^{2} \bigl\Vert A^{*}AX-XB^{*}B \bigr\Vert _{2}^{2} \bigr)^{\frac{1}{2}}, \end{aligned}$$
  *where* $r=\min\{p, 1-p\}$ *and* $r_{0}=\min\{2r, 1-2r\}$.

### Proof

The proof of inequality (17) is similar to that of inequality (16). Thus, we only need to prove the inequality (16).

If $0< p\leq\frac{1}{2}$, replacing *A* and *B* by $A^{*}A$ and $B^{*}B$ in inequality (8), respectively, we have

18 $$\begin{aligned} \bigl\Vert \bigl(A^{*}A\bigr)^{p}X \bigl(B^{*}B\bigr)^{1-p} \bigr\Vert _{2}&\leq \bigl( \bigl\Vert pA^{*}AX+(1-p)XB^{*}B \bigr\Vert _{2}^{2}-r_{0} \bigl\Vert \bigl(A^{*}A\bigr)^{\frac{1}{2}}X\bigl(B^{*}B \bigr)^{\frac {1}{2}}-A^{*}AX \bigr\Vert _{2}^{2} \\ &\quad {} -(1-p)^{2} \bigl\Vert A^{*}AX-XB^{*}B \bigr\Vert _{2}^{2} \bigr)^{\frac{1}{2}}. \end{aligned}$$

Interchanging the roles of *p* and $1-p$ in the inequality (18), we get

19 $$\begin{aligned} \bigl\Vert \bigl(A^{*}A\bigr)^{1-p}X \bigl(B^{*}B\bigr)^{p} \bigr\Vert _{2}&\leq \bigl( \bigl\Vert (1-p)A^{*}AX+pXB^{*}B \bigr\Vert _{2}^{2}-r_{0} \bigl\Vert \bigl(A^{*}A\bigr)^{\frac{1}{2}}X\bigl(B^{*}B \bigr)^{\frac{1}{2}}-A^{*}AX \bigr\Vert _{2}^{2} \\ &\quad {}-p^{2} \bigl\Vert A^{*}AX-XB^{*}B \bigr\Vert _{2}^{2} \bigr)^{\frac{1}{2}}. \end{aligned}$$

Applying inequalities (11), (18) and (19), we get the desired result. □

### Corollary 13

*Let* $A, B\in\mathcal{M}_{n}({\mathcal {C}})$ *and* $p\in(0, 1)$. *Then*

(i) *For* $0< p\leq\frac{1}{2}$,
  $$\begin{aligned} \bigl\Vert AB^{*} \bigr\Vert _{2}^{2} &\leq \bigl( \bigl\Vert pA^{*}A+(1-p)B^{*}B \bigr\Vert _{2}^{2}-r_{0} \bigl\Vert \bigl(A^{*}A\bigr)^{\frac{1}{2}}\bigl(B^{*}B \bigr)^{\frac{1}{2}}-A^{*}A \bigr\Vert _{2}^{2}\\ &\quad {} -(1-p)^{2} \bigl\Vert A^{*}A-B^{*}B \bigr\Vert _{2}^{2} \bigr)^{\frac {1}{2}} \\ &\quad {}\times \bigl( \bigl\Vert (1-p)A^{*}A+pB^{*}B \bigr\Vert _{2}^{2}-r_{0} \bigl\Vert \bigl(A^{*}A\bigr)^{\frac{1}{2}}\bigl(B^{*}B \bigr)^{\frac {1}{2}}-A^{*}A \bigr\Vert _{2}^{2}\\ &\quad {} -p^{2} \bigl\Vert A^{*}A-B^{*}B \bigr\Vert _{2}^{2} \bigr)^{\frac{1}{2}}. \end{aligned}$$
(ii) *For* $\frac{1}{2}< p<1$,
  $$\begin{aligned} \bigl\Vert AB^{*} \bigr\Vert _{2}^{2}&\leq \bigl( \bigl\Vert pA^{*}A+(1-p)B^{*}B \bigr\Vert _{2}^{2}-r_{0} \bigl\Vert \bigl(A^{*}A\bigr)^{\frac{1}{2}}\bigl(B^{*}B \bigr)^{\frac{1}{2}}-\bigl(B^{*}B\bigr) \bigr\Vert _{2}^{2}\\ &\quad {} -(1-p)^{2} \bigl\Vert A^{*}A-B^{*}B \bigr\Vert _{2}^{2} \bigr)^{\frac {1}{2}} \\ &\quad {}\times \bigl( \bigl\Vert (1-p)A^{*}A+pB^{*}B \bigr\Vert _{2}^{2}-r_{0} \bigl\Vert \bigl(A^{*}A\bigr)^{\frac{1}{2}}\bigl(B^{*}B \bigr)^{\frac {1}{2}}-\bigl(B^{*}B\bigr) \bigr\Vert _{2}^{2}\\ &\quad {} -p^{2} \bigl\Vert A^{*}A-B^{*}B \bigr\Vert _{2}^{2} \bigr)^{\frac{1}{2}}, \end{aligned}$$
  *where* $r=\min\{p, 1-p\}$ *and* $r_{0}=\min\{2r, 1-2r\}$.

Through the following, we would like to obtain upper bound for $\vert \!\vert \!\vert AXB^{*} \vert \!\vert \!\vert $, for every unitary invariant norm.

The following lemma has been shown in [16], and it is considered as a refined matrix Young inequality for every unitary invariant norm.

### Lemma 14

*Let* $A, B, X\in\mathcal{M}_{n}$ *such that* *A*, *B* *are positive semidefinite*. *Then*, *for* $0\leq p\leq1$, *we have*

20 $$\begin{aligned} \bigl\vert \!\bigl\vert \!\bigl\vert A^{p}XB^{1-p} \bigr\vert \!\bigr\vert \!\bigr\vert ^{2}+r_{0}\bigl( \vert \!\vert \!\vert AX \vert \!\vert \!\vert - \vert \!\vert \!\vert XB \vert \!\vert \!\vert \bigr)^{2}\leq\bigl(p \vert \!\vert \!\vert AX \vert \!\vert \!\vert +(1-p) \vert \!\vert \!\vert XB \vert \!\vert \!\vert \bigr)^{2}, \end{aligned}$$

*where* $r_{0}=\min\{p,1-p\}$.

### Proposition 15

*Let* $A, B, X\in\mathcal{M}_{n}$. *Then*

$$\begin{aligned} \bigl\vert \!\bigl\vert \!\bigl\vert AXB^{*} \bigr\vert \!\bigr\vert \!\bigr\vert ^{2}&\leq \bigl(\bigl(p \bigl\vert \!\bigl\vert \!\bigl\vert A^{*}AX \bigr\vert \!\bigr\vert \!\bigr\vert +(1-p) \bigl\vert \!\bigl\vert \!\bigl\vert XB^{*}B \bigr\vert \!\bigr\vert \!\bigr\vert \bigr)^{2}-r_{0}^{2} \bigl( \bigl\vert \!\bigl\vert \!\bigl\vert A^{*}AX \bigr\vert \!\bigr\vert \!\bigr\vert - \bigl\vert \!\bigl\vert \!\bigl\vert XB^{*}B \bigr\vert \!\bigr\vert \!\bigr\vert \bigr)^{2} \bigr)^{\frac{1}{2}} \\ &\quad {}\times \bigl(\bigl((1-p) \bigl\vert \!\bigl\vert \!\bigl\vert A^{*}AX \bigr\vert \!\bigr\vert \!\bigr\vert +p \bigl\vert \!\bigl\vert \!\bigl\vert XB^{*}B \bigr\vert \!\bigr\vert \!\bigr\vert \bigr)^{2}-r_{0}^{2}\bigl( \bigl\vert \!\bigl\vert \!\bigl\vert A^{*}AX \bigr\vert \!\bigr\vert \!\bigr\vert - \bigl\vert \!\bigl\vert \!\bigl\vert XB^{*}B \bigr\vert \!\bigr\vert \!\bigr\vert \bigr)^{2} \bigr)^{\frac{1}{2}}, \end{aligned}$$

*where* $p\in[0,1]$ *and* $r_{0}=\min\{p,1-p\}$.

### Proof

In inequality (20), if we replace *A* by $A^{*}A$ and *B* by $B^{*}B$, then we have

21 $$\begin{aligned} \bigl\vert \!\bigl\vert \!\bigl\vert \bigl(A^{*}A\bigr)^{p}X\bigl(B^{*}B \bigr)^{1-p} \bigr\vert \!\bigr\vert \!\bigr\vert &\leq \bigl(\bigl(p \bigl\vert \!\bigl\vert \!\bigl\vert A^{*}AX \bigr\vert \!\bigr\vert \!\bigr\vert +(1-p) \bigl\vert \!\bigl\vert \!\bigl\vert XB^{*}B \bigr\vert \!\bigr\vert \!\bigr\vert \bigr)^{2} \\ &\quad {}-r_{0}^{2}\bigl( \bigl\vert \!\bigl\vert \!\bigl\vert A^{*}AX \bigr\vert \!\bigr\vert \!\bigr\vert - \bigl\vert \!\bigl\vert \!\bigl\vert XB^{*}B \bigr\vert \!\bigr\vert \!\bigr\vert \bigr)^{2} \bigr)^{\frac{1}{2}}. \end{aligned}$$

Interchanging *p* with $1-p$ in inequality (21), we get

22 $$\begin{aligned} \bigl\vert \!\bigl\vert \!\bigl\vert \bigl(A^{*}A\bigr)^{1-p}X\bigl(B^{*}B \bigr)^{p} \bigr\vert \!\bigr\vert \!\bigr\vert &\leq \bigl(\bigl((1-p) \bigl\vert \!\bigl\vert \!\bigl\vert A^{*}AX \bigr\vert \!\bigr\vert \!\bigr\vert +p \bigl\vert \!\bigl\vert \!\bigl\vert XB^{*}B \bigr\vert \!\bigr\vert \!\bigr\vert \bigr)^{2} \\ &\quad {}-r_{0}^{2}\bigl( \bigl\vert \!\bigl\vert \!\bigl\vert A^{*}AX \bigr\vert \!\bigr\vert \!\bigr\vert - \bigl\vert \!\bigl\vert \!\bigl\vert XB^{*}B \bigr\vert \!\bigr\vert \!\bigr\vert \bigr)^{2} \bigr)^{\frac{1}{2}}. \end{aligned}$$

Now applying inequalities (11), (21) and (22) we get the desired inequality. □

## Conclusions

Our application of the methods based on the Audenaert results is presented in this paper to the operator norm and so are some interpolations for an arbitrary unitarily invariant norm. Moreover, we refine some previous inequalities as regards the Cauchy-Schwarz inequality for the operator and Hilbert-Schmidt norms.
